# A Prediction Model Using Alternative Splicing Events and the Immune Microenvironment Signature in Lung Adenocarcinoma

**DOI:** 10.3389/fonc.2021.778637

**Published:** 2021-12-22

**Authors:** Liping Zhu, Zhiqiang Wang, Yilan Sun, Georgios Giamas, Justin Stebbing, Zhentao Yu, Ling Peng

**Affiliations:** ^1^ Department of Medical Oncology, Shouguang Hospital of Traditional Chinese Medicine, Shouguang, China; ^2^ Department of Urology, Shouguang Hospital of Traditional Chinese Medicine, Shouguang, China; ^3^ Department of Respiratory Disease, Zhejiang Provincial People’s Hospital, Hangzhou, China; ^4^ Department of Biochemistry and Biomedicine, School of Life Sciences, University of Sussex, Brighton, United Kingdom; ^5^ Division of Cancer, Department of Surgery and Cancer, Imperial College London, London, United Kingdom; ^6^ Department of Thoracic Surgery, Shenzhen Hospital, Southern Center, National Cancer Center, Shenzhen, China

**Keywords:** lung cancer, lung adenocarcinoma, alternative splicing, prognosis, tumor immune microenvironment, immunotherapy

## Abstract

**Background:**

Alternative splicing (AS) is a gene regulatory mechanism that drives protein diversity. Dysregulation of AS is thought to play an essential role in cancer initiation and development. This study aimed to construct a prognostic signature based on AS and explore the role in the tumor immune microenvironment (TIME) in lung adenocarcinoma.

**Methods:**

We analyzed transcriptome profiling and clinical lung adenocarcinoma data from The Cancer Genome Atlas (TCGA) database and lists of AS-related and immune-related signatures from the SpliceSeq. Prognosis-related AS events were analyzed by univariate Cox regression analysis. Gene set enrichment analyses (GSEA) were performed for functional annotation. Prognostic signatures were identified and validated using univariate and multivariate Cox regression, LASSO regression, Kaplan–Meier survival analyses, and proportional hazards model. The context of TIME in lung adenocarcinoma was also analyzed. Gene and protein expression data of Cyclin-Dependent Kinase Inhibitor 2A (CDKN2A) were obtained from ONCOMINE and Human Protein Atlas. Splicing factor (SF) regulatory networks were visualized.

**Results:**

A total of 19,054 survival-related AS events in lung adenocarcinoma were screened in 1,323 genes. Exon skip (ES) and mutually exclusive exons (ME) exhibited the most and fewest AS events, respectively. Based on AS subtypes, eight AS prognostic signatures were constructed. Patients with high-risk scores were associated with poor overall survival. A nomogram with good validity in prognostic prediction was generated. AUCs of risk scores at 1, 2, and 3 years were 0.775, 0.736, and 0.759, respectively. Furthermore, the prognostic signatures were significantly correlated with TIME diversity and immune checkpoint inhibitor (ICI)-related genes. Low-risk patients had a higher StromalScore, ImmuneScore, and ESTIMATEScore. AS-based risk score signature was positively associated with CD8+ T cells. CDKN2A was also found to be a prognostic factor in lung adenocarcinoma. Finally, potential functions of SFs were determined by regulatory networks.

**Conclusion:**

Taken together, our findings show a clear association between AS and immune cell infiltration events and patient outcome, which could provide a basis for the identification of novel markers and therapeutic targets for lung adenocarcinoma. SF networks provide information of regulatory mechanisms.

## Introduction

Lung cancer is the leading cause of cancer death worldwide, with non-small cell lung cancer (NSCLC) being the most prevalent type (1). In recent years, targeted therapies and immunotherapies have brought unprecedented clinical benefits to lung cancer patients. Immune checkpoint inhibitors (ICIs) have emerged as a new strategy for the treatment of lung cancer and in combination with other anti-cancer therapies, such as chemotherapy and anti-angiogenesis drugs; they have increased the effectiveness of therapeutic regimens.

The pivotal role of the tumor microenvironment (TME) in the tumorigenesis and progression of lung cancer has been well established. The immune cells in the lung TME harbor both pro-tumor and anti-tumor activities, which can help predict clinical outcome. The positive effects of ICIs are easier to detect in individual cancer patients, as intratumoral heterogeneity may influence the anti-tumor immune response. Therefore, it is of relevance to identify biomarkers that have prognostic value in stratifying patients.

Alternative splicing (AS) of precursor mRNAs represents a major mechanism in the maturation of mRNAs (2). AS enables one gene to encode an array of proteins. AS contributes to posttranscriptional gene regulation, which functions in physiological and pathological processes, while dysregulated AS events participate in tumor development and metastasis (3, 4). The dysregulated expressed genes could serve as a prognostic biomarker and therapeutic target. Splicing factors (SFs) bind to gene-specific splice-regulatory sequence elements and comprise a regulatory network (5, 6). Albeit aberrant, AS can transform normal cells into malignant ones (6–8); still, its role on tumorigenesis remains largely unknown. Thus, investigation on dysregulated AS network may provide information on prognostic biomarkers for cancer treatment (9–11). There have been several studies investigating prognostic biomarkers based on AS events (12–14); however, the relationship of AS prognostic signatures with the tumor immune microenvironment (TIME) is lacking.

In this study, the AS pattern of a TCGA-lung adenocarcinoma (LUAD) cohort was delineated, and survival-associated AS events were identified. Following, AS-based prognostic signatures were constructed and validated. An AS-clinicopathologic nomogram was generated to inform clinical decision-making. Moreover, the relationship of prognostic signatures with TIME was explored, while the role of CKDN2A in lung adenocarcinoma was further investigated. Finally, an AS-SFs regulatory network was constructed to demonstrate the potential mechanism of lung adenocarcinoma progression.

## Methods and Materials

### Multiomics Data Acquisition

Data of transcriptome information and survival of lung adenocarcinoma patients were retrieved from The Cancer Genome Atlas (TCGA) (http://cancergenome.nih.gov). Data of AS of the TCGA LUAD-cohort were downloaded from SpliceSeq (http://bioinformatics.mdanderson.org/TCGASpliceSeq). Samples were selected if PSI (Percentage Of Spliced In) value > 75% as filter threshold. The flow chart of analysis was presented in **Supplementary Figure S1**.

### AS Profile Identification

The PSI values were calculated to quantify AS events. There are 7 subtypes of AS events delineated using Upset plot, e.g., alternate acceptor site (AA), alternate donor site (AD), alternate promoter (AP), alternate terminator (AT), exon skip (ES), mutually exclusive exons (ME), and retained intron (RI). The splicing type, ID number in the SpliceSeq, and the corresponding parent gene symbol were used to annotate AS events. For example, in “XAF1|38812|AA”, XAF1 indicates the corresponding parent gene name, 38812 denotes the ID of splicing variant, and AA represents the splicing type.

### Identification of Survival-Related AS Events

The AS data were excluded if the standard deviation of PSI value < 1%. Univariate Cox regression analysis was performed to analyze the association between AS events and overall survival of lung adenocarcinoma patients (**Supplementary File: Table S1**). The top 20 most significant AS events of different subtypes were displayed.

### Construction and Validation of Prognostic Signature

Firstly, candidates in each splicing pattern were detected using Lasso regression analysis. Secondly, selected AS events were submitted to Multivariate Cox regression analysis. The identified AS events in each splicing subtype were integrated to construct another prognostic signature. Then, risk scores were calculated based on each prognostic predictor. The formula to calculate the risk score is as follows:


$$Risk\;score=\sum_{i=1}^{n}Coef_{i}\times PSI_{i}$$

Where Coef_i_ means the coefficients and PSI_i_ is the percent-spliced-in value of each AS. Patients were separated into a low-risk group and a high-risk group based on the median value of risk score. Kaplan–Meier survival curves were analyzed. Then, the time-dependent receiver operating characteristic (ROC) curves were generated. Univariate and multivariate Cox regression were performed to determine whether the signature can act as an independent factor for prognostic prediction. Stratified survival analysis was performed to validate the prognostic capability independent from clinical characteristics.

### Construction of a Prognostic Nomogram

To investigate the prognosis predictive ability of risk signature, age, grade, tumor stage, T/N/M category for 1-, 2-, and 3-year overall survival, and time-dependent ROC curves were performed to calculate the AUC (area under the curve) values. A nomogram was established to estimate 1-, 2-, and 3- year overall survival probability. The calibration curve was delineated.

### Correlation of Risk Score With Infiltrating Immune Cells in TIME

Information on immune infiltration and immune cell fraction were retrieved from tumor immune estimation resource (TIMER) (https://cistrome.shinyapps.io/timer/). The correlation between infiltration of immune cell with the prognostic risk score was performed. A single sample gene-set enrichment analysis (ssGSEA) was performed to investigate the enrichment of the two different risky subgroups in 29 immune function-associated gene sets. Subsequently, tumor purity and the extent and level of infiltrating cells were assessed. The fraction of 22 immune cell types for each tumor specimen was developed through CIBERSORT (https://cibersort.stanford.edu/).

### Role of Risk Score in Immune Checkpoint Blockade Treatment

Herein, 6 key genes of ICI in lung adenocarcinoma were extracted, e.g., programmed death ligand 1 (PD-L1 or CD274), programmed death ligand 2 (PD-L2, or PDCD1LG2), programmed death 1 (PD-1, or PDCD1), cytotoxic T-lymphocyte antigen 4 (CTLA-4), T-cell immunoglobulin domain and mucin domain-containing molecule-3 (TIM-3, or HAVCR2), and indoleamine 2,3-dioxygenase 1 (IDO1). To investigate the potential role of as-constructed risk signature in ICI treatment of lung adenocarcinoma, AS-based prognostic signature and expression level of 6 ICI key genes were correlated. Finally, expression level of 47 ICI-related genes between low- and high-risk groups were compared.

### Gene and Protein Expression Data

Information on gene expression were obtained from the ONCOMINE website (https://www.oncomine.org/). Tumor type was lung adenocarcinoma, and the expressions of CDKN2A were obtained. The levels of CDKN2A between lung adenocarcinoma specimens and normal controls were analyzed by online tools. The protein expression level of CDKN2A was verified by The Human Protein Atlas (https://www.proteinatlas.org/).

### Construction of Splicing Regulatory Network

A list of 404 splicing factors (SFs) reported by a previous study (7) was shown in **Supplementary File: Table S2**. The RNA-seq profiles of SFs were retrieved from the TCGA database. The association between SFs and survival-relevant AS events were investigated by Spearman correlation analysis. The cutoff values were *p* < 0.001 and correlation coefficient > 0.6. Finally, Cytoscape (version 3.8.0) was used to build an SF-AS regulatory network.

### Statistical Analysis

The Wilcoxon test was performed to compare two groups, whereas the Kruskal–Wallis test was used to compare more than two groups. Risk scores, clinical variables, immune cell infiltration, and immune checkpoints were correlated with Pearson correlation test. *p* < 0.05 was considered as statistical significance. Perl software (version 6.1.7601) was used to perform expression analysis. R software (version 4.0.3) was used for all statistical analyses.

## Results

### Clinical Characteristics and Integrated AS Events Profiles in Lung Adenocarcinoma

The profiles of AS events/genes of 572 TCGA-LUAD patients were obtained, consisting of 513 tumor samples and 59 corresponding normal samples. A total of 551 lung adenocarcinoma patients were obtained using the TCGA database, and 65 patients with incomplete information were excluded, with 486 patients enrolled.

**Figure 1:**
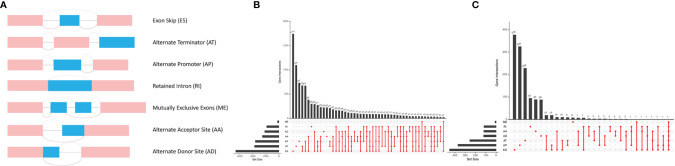
Different AS types in lung adenocarcinoma. **(A)** Seven different AS types of genes. **(B)** Upset plot of interactions between different AS types in lung adenocarcinoma. **(C)** Upset plot of different survival-associated AS types.

In total, 43,948 splicing events were detected in 10,005 genes using SpliceSeq. The different AS events were classified into 7 types: ES, AA, AP, AD, AT, ME, and RI, which are illustrated in **Figure 1A**. ES and AT events are the most frequent. The interaction numbers between genes and different AS classes are shown in **Figure 1B**. ES is the highest AS events in number, while ME is the rarest.

### Identification of the Survival-Relevant AS Events

**Figure 2:**
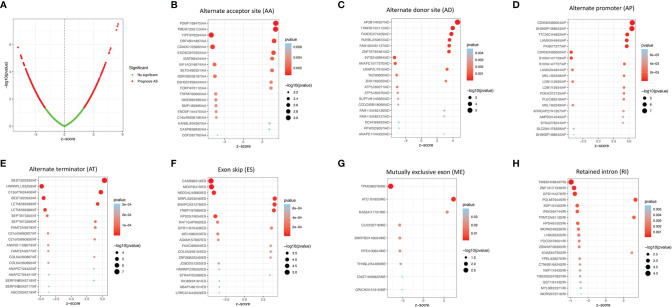
The survival-relevant AS events. **(A)** Volcano plots of survival-relevant AS events. The most significant survival-relevant alternate acceptor site **(B)**, alternate donor site **(C)**, alternate promoter **(D)**, alternate terminator **(E)**, exon skip **(F)**, mutually exclusive exons **(G)**, and retained intron **(H)** in the TCGA-LUAD cohort.

A univariate Cox proportional hazards regression analysis was performed by the Perl language (http://www.perl.org/). A total of 19,054 survival-associated AS events were detected in 1,323 genes. The interaction between these genes and different AS types is shown in **Figure 1C**. Eight AS events (AA, AD, AP, AT, ES, ME, RI, and ALL) were associated with overall survival in lung adenocarcinoma patients. The AS events (**Supplementary Table S3**) were displayed in a volcano plot (**Figure 2A**). The top 20 significant survival-related AS events from the 7 subtypes are summarized in **Figures 2B–H**. Among all the AS events, PSMF1|58475|AA, AP2B1|40327|AD, CDKN2A|86004|AP, BEST3|23330|AT, CA5B|98313|ES, TPM2|86278|ME, and TMSB4X|88497|RI were the most significant events for AA, AD, AP, AT, ES, ME, and RI, respectively.

### Confirmation of the Prognostic Signature

**Figure 3:**
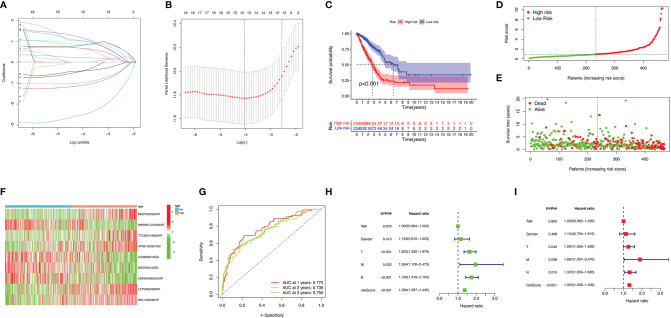
Confirmation of ALL AS-based prognostic signature. **(A)** LASSO coefficient profiles of the whole AS events. **(B)** Ten-time cross-validation for tuning parameter selection in Lasso regression. **(C)** Kaplan–Meier curve of survival in the high- and low-risk cohorts. **(D)** Heatmap of the ALL signature AS events PSI value in lung adenocarcinoma. The color from red to green shows a trend from high to low expression. **(E)** Distribution of ALL signature risk score. **(F)** Heatmap of the ALL signature AS events PSI value in lung adenocarcinoma. **(G)** ROC analysis of ALL risk scores for overall survival prediction. Univariate **(H)** and multivariate **(I)** Cox regression results.

Eight AS (AA, AD, AP, AT, ES, ME, RI, and ALL) prognostic signatures were constructed. Lung adenocarcinoma patients were stratified into low- and high-risk subgroups based on the cutoff value of median risk score. Lasso plot (**Figure 3A**) and Lambda plot (**Figure 3B**) were performed to avoid overfitting. Finally, 9 AS were selected for multivariate Cox regression analysis, namely, HNRNPLL|53258|AT, CA5B|98313|ES, MEGF6|315|ES, CDKN2A|86000|AP, BEST3|23330|AT, TTC39C|44852|AP, AP2B1|40327|AD, LETM2|83399|AT, and MKL1|62348|AP (**Table 1**). The results (**Figure 3C**) show the survival probability of each group, indicating a significant difference between them. Kaplan–Meier curve demonstrates the reliability of the model with a *p*-value < 0.001. The risk curve (**Figure 3D**) and scatterplot (**Figure 3E**) indicate that high-risk lung adenocarcinoma patients have a shorter overall survival. The heatmap reveals that HNRNPLL|53258|AT, CA5B|98313|ES, MEGF6|315|ES, and CDKN2A|86000|AP may have positive effects on lung adenocarcinoma while BEST3|23330|AT, TTC39C|44852|AP, AP2B1|40327|AD, LETM2|83399|AT, and MKL1|62348|AP can have adverse effects (**Figure 3F**).

**Table 1:** Nine AS events selected for multivariate analysis.

ID	Coefficient	Hazard Ratio	95% Lower Limit	95% Upper Limit	*p*-value
BEST3|23330|AT	1.390212	4.015703	1.374436	11.73272	0.011042
HNRNPLL|53258|AT	−2.72959	0.065246	0.007077	0.601561	0.016023
TTC39C|44852|AP	0.94922	2.583693	1.033915	6.456501	0.042221
AP2B1|40327|AD	1.098875	3.000787	0.669683	13.44624	0.151001
CA5B|98313|ES	−1.28079	0.277817	0.105584	0.731003	0.009466
MEGF6|315|ES	−1.42567	0.240347	0.097459	0.592726	0.001964
CDKN2A|86000|AP	−1.36077	0.256463	0.121138	0.542963	0.000377
LETM2|83399|AT	1.329962	3.780902	1.302816	10.97255	0.014422
MKL1|62348|AP	1.843278	6.317212	1.65184	24.15922	0.007075

ROC curve was analyzed to investigate the prognostic value of risk signatures in lung adenocarcinoma. AUCs of risk scores at 1, 2, and 3 years were 0.775, 0.736, and 0.759, respectively, suggesting good sensitivity and specificity of the survival predictive ability (**Figure 3G**). Univariate and multivariate Cox regression analyses were applied to age, gender, stage, TNM stage, and risk score. With both *p*-values < 0.001 of the risk score in two analyses and hazard ratios of 1.364 [95% confidence interval (CI): 1.287–1.445] and 1.320 (1.238–1.409), the risk score proved to be a robust model (**Figures 3H, I**). Consequently, SFs can act as a predictor for survival.

### Correlation of ALL Prognostic Signature With Clinical Features and Construction of AS-Clinicopathological Nomogram

**Figure 4:**
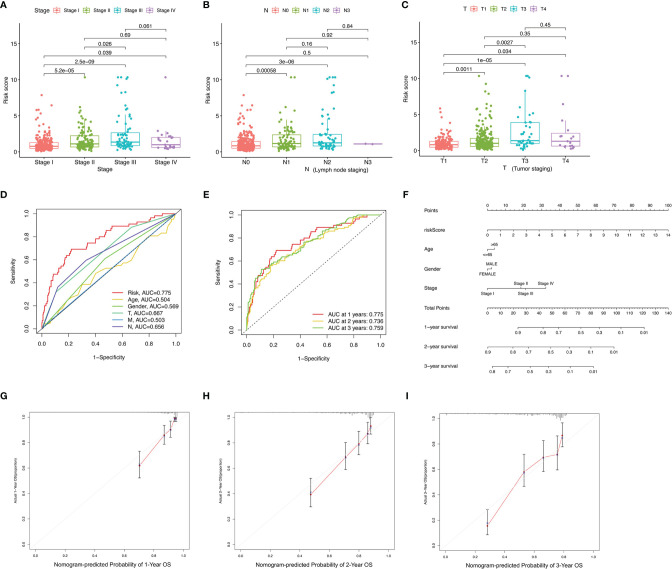
Correlation of prognostic signature with clinical features and construction of AS-clinicopathological nomogram. **(A–E)** Correlation of risk score with stage **(A)**, N status **(B)**, and T status **(C)**. **(D, E)** AUCs for predicting 1-, 2-, and 3-year survival with different clinical characteristics. **(F)** Nomogram was assembled for predicting survival of lung adenocarcinoma patients. **(G–I)** 1-, 2-, and 3-year nomogram calibration curves.

Differences of risk score among clinical variables were explored. The risk score increased significantly with tumor grade (most *p* < 0.05, **Figure 4A**), N category (most *p* < 0.05, **Figure 4B**), and T category (most *p* < 0.05, **Figure 4C**), suggesting ALL risk score to be positively correlated with tumor progression. To investigate whether ALL prognostic signature was the best prognostic indicator among clinical characteristics, several parameters were extracted as potential prognosis/predictive factors, such as age, gender, clinicopathological stage, and tumor grade. These clinical parameters were combined to conduct AUC curve analysis for 1-, 2-, and 3-year overall survival, and risk signature had the most AUC value (**Figures 4D, E**). Then, prognostic nomogram including risk score and clinicopathological stage were constructed to predict prognosis of lung adenocarcinoma patients (**Figure 4F**). Age, gender, and tumor grade were rejected out of the nomogram, because of their AUCs being < 0.6. Calibration curves were approximately diagonal, indicating robust ability of informing prognosis (**Figures 4G–I**).

### Correlation of Risk Score With TIME Characterization

To further examine whether risk score can act as an immune indicator, correlation analyses of prognostic risk score with tumor-infiltrating immune cells (TICs) from TIMER, StromalScore, ImmuneScore and ESTIMATEScore (ESTIMATE algorithm), ssGSEA signatures, and TICs subtype and level (CIBERSORT method) were performed. The higher score estimated in ImmuneScore or StromalScore were represented for the larger amount of the immune or stromal components in TME. ESTIMATEScore was the sum of ImmuneScore and StromalScore, indicating the comprehensive proportion of both components in the TME (15).

**Figure 5:**
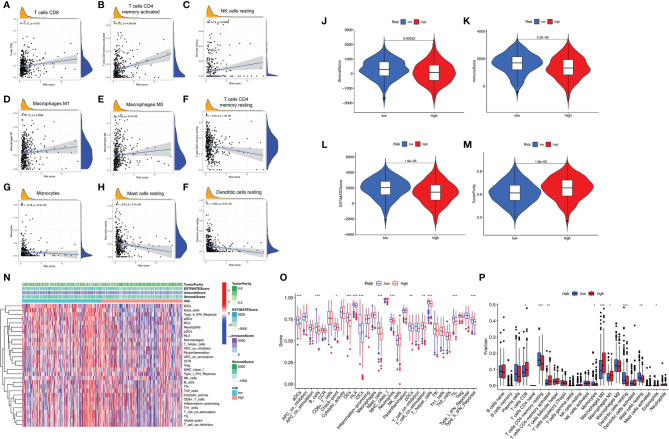
Correlation between infiltrating immune cells and AS-based prognostic signature. **(A–F)** Relationship between this signature and CD8-positive T cells **(A)**, T cells CD4 memory activated **(B)**, NK cells resting **(C)**, macrophages M1 **(D)**, macrophages M0 **(E)**, T cells CD4 memory resting **(F)**, monocytes **(G)**, mast cells resting **(H)**, and dendritic cells resting **(I)**. **(J–M)** Comparison of StromalScore **(J)**, ImmuneScore **(K)**, ESTIMATEScore **(L)**, and tumor purity **(M)** between low- and high-risk groups, respectively. **(N)** Red represent high activity, and blue represents low activity. **(O)** Distinct enrichment of immune-related signatures between low- and high-risk groups. **(P)** Difference of infiltrating immune cell subpopulations and levels between low- and high-risk groups. *p <0.05, **p <0.005, ***p <0.001.

Firstly, TIMER results showed that the as-constructed signature exhibited positive association with CD8+ T cells (*r* = 0.11; *p* = 0.02), activated CD4+ memory T cells (*r* = 0.2; *p* = 4.5e−05), resting NK cells (*r* = 0.13; *p* = 0.0066), M1 macrophages (*r* = 0.13; *p* = 0.0096), and M0 macrophages (*r* = 0.28; *p* = 6.7e−09; **Figures 5A–E**), indicating that high-risk samples were infiltrated by more immune cells. A negative correlation was observed between risk score and infiltration levels of the resting CD4+ memory T cells (*r* = −0.23; *p* = 1.4e−06), monocytes (*r* = −0.19; *p* = 6.5e−05), resting mast cells (*r* = −0.23; *p* = 2.7e−06), and resting dendritic cells (*r* = −0.26; *p* = 6.2e−08; **Figures 5F–I**). Likewise, low-risk patients had a higher StromalScore, ImmuneScore, and ESTIMATEScore (**Figures 5J–L**). There was higher tumor purity in high-risk patients that represented less ESTIMATEScore (**Figure 5M**). Subsequently, distinction of the immune-related signatures between two subgroups was presented. **Figure 5N** shows the immune-related signature of each patient with corresponding ImmuneScore in low- and high-risk groups. The results revealed that the infiltration of activated dendritic cells (aDCs), B-cells, checkpoint, DCs, HLA, immature DCs (iDCs), mast cells, neutrophils, pDCs, T-cell co-stimulation, T helper cells, TIL (tumor-infiltrating lymphocytes), and type IFN response are significantly decreased with increased risk score (**Figure 5O**). CIBERSORT algorithm results indicated that the proportion of CD8+ T cells and M0 and M1 macrophages are positively associated with risk score. However, the proportion of resting CD4+ memory T cells, monocytes, resting dendritic cells, and resting mast cells are negatively associated with risk score (**Figure 5P**). The above results revealed that ALL prognostic signature may provide a novel approach to elucidate the characteristics of immunity regulatory network in lung adenocarcinoma.

### Correlation of ALL Signature with ICI Key Molecules

**Figure 6:**
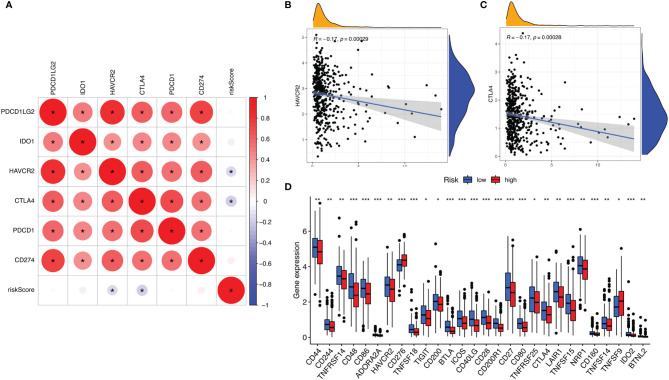
**(A)** Association analyses between 6 key immune checkpoints and risk score. Association between risk score with HAVCR2 **(B)** and CTLA4 **(C)**. **(D)** Comparison of ICI-related genes expression levels between low- and high-risk groups. *p <0.05, **p <0.005, ***p <0.001.

ICIs have considerably transformed clinical decision-making in cancer oncology. In our study, 6 key ICI genes (PDCD1, CD274, PDCD1LG2, CTLA-4, HAVCR2, and IDO1) (16) were evaluated. The correlation between ICI key targets and ALL prognostic signature was analyzed to investigate the potential role of risk signature in ICI treatment of lung adenocarcinoma (**Figure 6A**). The results indicated that ALL prognostic signature is negatively correlated to HAVCR2 (*r* = -0.17; *p* = 0.00029) and CTLA4 (*r* = -0.17; *p* = 0.00028; **Figures 6B, C**). Further correlation analysis suggested that 28 of 47 ICI-associated genes’ expression levels are significantly upregulated in patients with low risk (**Figure 6D**), suggesting that ALL prognostic signature may serve as an unfavorable factor in immunotherapy treatment.

### CDKN2A Independently Affected Prognosis and Correlated With ICI Key Genes

**Figure 7:**
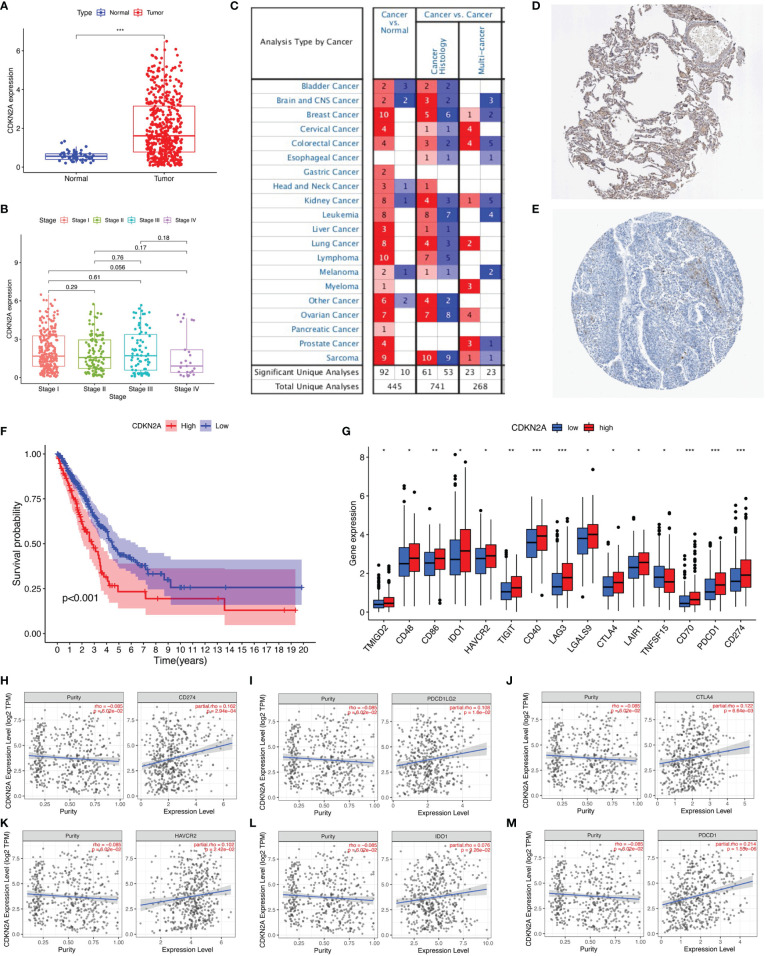
The clinical significance of CDKN2A in lung adenocarcinoma. **(A)** CDKN2A is higher expressed in lung adenocarcinoma tumor tissue. **(B)** No significant correlation of CDKN2A with tumor grade. **(C)** Analyses from ONCOMINE website show high expression of CDKN2A. **(D, E)** The protein expression level of CDKN2A was verified by The Human Protein Atlas. **(F)** Lower CDKN2A level predicts longer overall survival. **(G)** Comparison of ICI-related genes’ expression levels between low- and high-CDKN2A group. **(H–M)** Correlation of risk score with CD274 **(H)**, PDCD1LG2 **(I)**, CTLA4 **(J)**, HAVCR2 **(K)**, IDO1 **(L)**, and PDCD1 **(M)**. *p <0.05, **p <0.005, ***p <0.001.

CDKN2A was the only gene whose expression was upregulated among the prognostic AS-related genes. Therefore, the role of CDKN2A in lung adenocarcinoma was further explored. CDKN2A expression levels between normal tissues and tumor samples were compared using the TCGA data. Relative to tumor tissues, the expression level of CDKN2A was lower in adjacent normal specimens (**Figure 7A**). Analysis among major pathological stages suggested that there is no significant difference of CDKN2A expression levels among different stages (**Figure 7B**). High expression has been detected in different cancers according to the ONCOMINE website (**Figure 7C**), while the protein expression l of CDKN2A was verified, as shown in The Human Protein Atlas (**Figures 7D, E**). Kaplan–Meier analyses were conducted between CDKN2A low- and high-expressed patients. Lower CDKN2A expression levels suggested longer overall survival period (*p* < 0.001, **Figure 7F**). Moreover, 15 of 47 ICI-associated genes’ expression levels between low- and high-CDKN2A groups were significantly dysregulated between subgroups (**Figure 7G**). The correlation between CDKN2A and ICI key targets adjusted by tumor purity using TIMER was analyzed to investigate the potential role of CDKN2A in ICI treatment of lung adenocarcinoma. TIMER results revealed that CDKN2A is positively correlated with CD274 (*r* = 0.162; *p* = 2.94e−04), PDCD1LG2 (*r* = 0.108; *p* = 1.6e−02), CTLA4 (*r* = 0.122; *p* = 6.64e−03), HAVCR2 (*r* = 0.102; *p* = 2.42e−02), IDO1 (*r* = 0.076; *p* = 9.26e−02), and PDCD1 (*r* = 0.213; *p* = 1.55e−06; **Figures 7H–M**), suggesting that CDKN2A plays a role in ICI treatment of lung adenocarcinoma.

### Role of CDKN2A in Context of TIME

**Figure 8:**
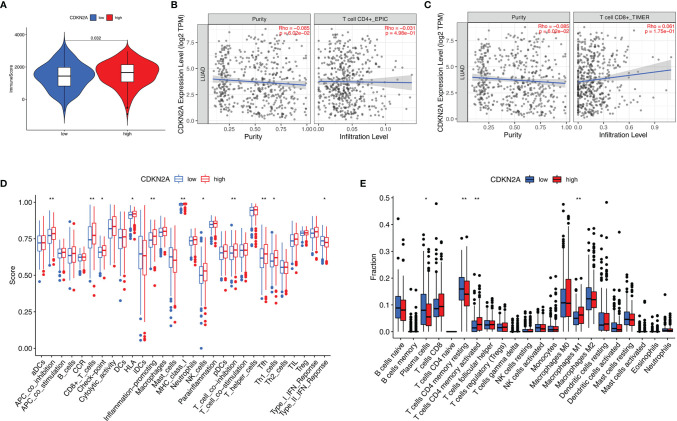
The role of CDKN2A in TIME features. **(A)** Comparison of ImmuneScore between low- and high-CDKN2A groups. **(B, C)** Relationship between risk score with CD4 T cells **(B)** and CD8 T cells **(C)**. **(D, E)** Comparison of ssGSEA enrichment **(D)** and CIBERSORT results **(E)** between low- and high-CDKN2A groups. *p <0.05, **p <0.005.

To further investigate the relationship between CDKN2A and TIME characteristics in lung adenocarcinoma, comprehensive analyses were performed. Lung cancer patients were separated into high- and low-CDKN2A subtypes based on the median CDKN2A expression levels. ESTIMATE results indicated that patients with high-CDKN2A expression have a significantly higher ImmuneScore compared with patients in low- CDKN2A group, suggesting the presence of fewer immune cells in low-risk samples (**Figure 8A**). Subsequently, expression levels of CDKN2A were negatively correlated with infiltration of CD4+ T cells (**Figure 8B**) and positively correlated with infiltration of CD8+ T cells (**Figure 8C**). Results of ssGSEA revealed that the infiltration fraction of APC co-inhibition, CD8+ T cells, checkpoint, HLA, inflammation promoting, MHC-class, NK cells, T cell co-inhibition, Tfh (T follicular helper cell), and Th1 cell expression are significantly decreased when risk score declines (**Figure 8D**). CIBERSORT analysis results showed that the proportion of plasma cells and resting CD4+ memory T cells are significantly higher in low-risk patients and the proportion of M1 macrophages and activated CD4+ memory T cells are significantly higher in high-risk patients (**Figure 8E**).

### Development of the SF-AS Regulatory Network

**Figure 9:**
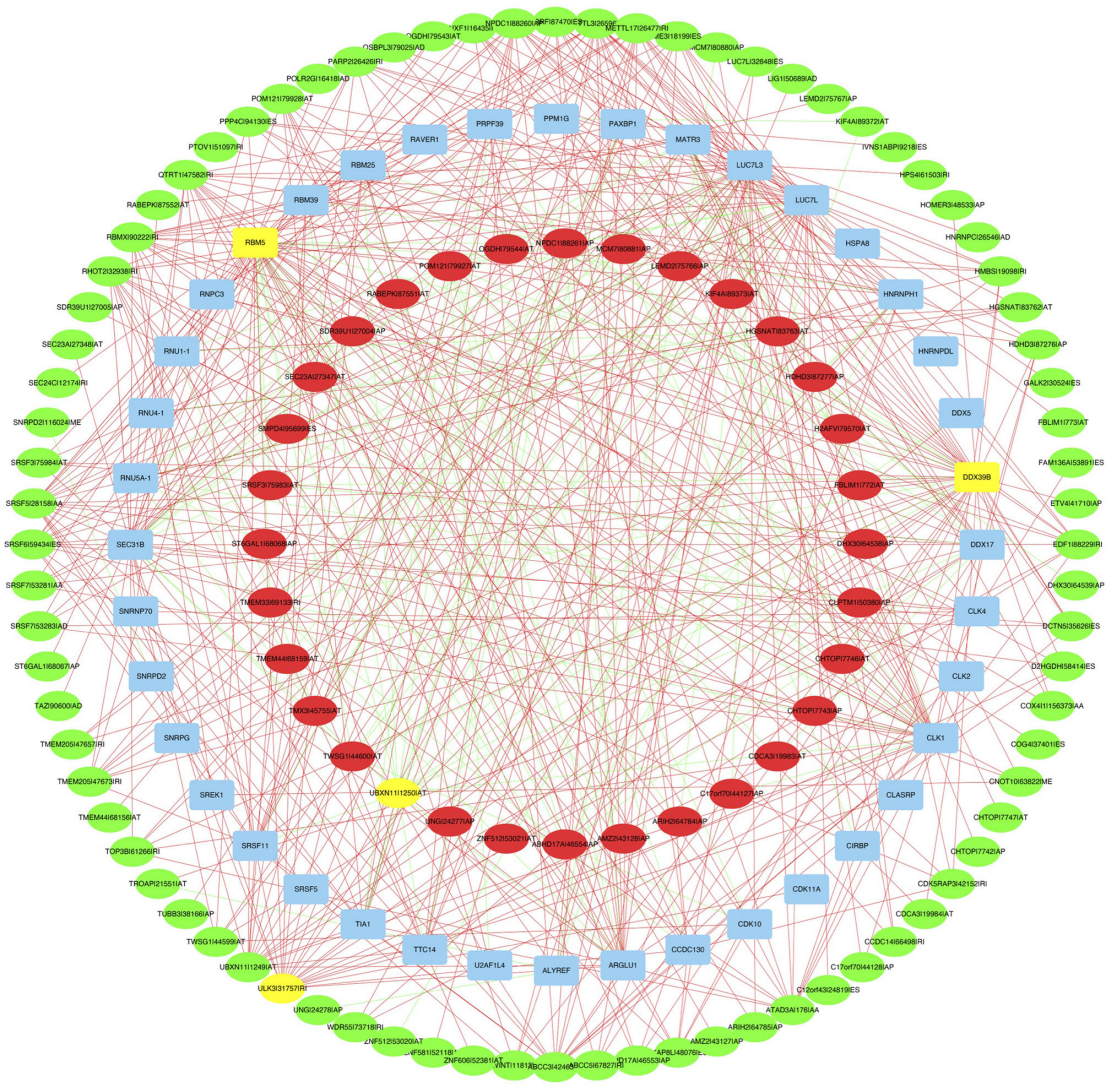
The regulatory network between SFs and survival related AS events. The red or green ellipses indicate AS events that positively or negatively correlate with survival (red represents high-risk AS, green represents low-risk AS). Blue ellipses represent SFs. The positive/negative correlations (*r* > 0.8 or *r* < −0.8) between SFs and AS events are indicated with red/green lines. The top 4 most significant nodes were highlighted in yellow.

A correlation network between the expression levels of SFs and the PSI values of prognosis-related AS events was constructed. Thirty-two upregulated AS events (red ellipses), 80 downregulated AS events (green ellipses), and 40 SFs (**Figure 9**) were identified. In our regulatory network, the top 4 most significant nodes were termed hub SFs or AS events (**Supplementary Table S4**), including 1 downregulated AS event (ULK3|31757|RI), 1 upregulated AS event (UBXN11|1250|AT), and 2 SFs (DDX39B and RBM5). Therefore, these SFs exhibited potential to act as regulators, which was involved in the dysregulation of AS in lung adenocarcinoma.

## Discussion

In our study, AS data were retrieved from TCGA SpliceSeq and 19,054 survival-associated AS events were identified. Then, prognostic signatures for lung adenocarcinoma patients were constructed. Eight AS events’ (AA, AD, AP, AT, ES, ME, RI, and ALL) signatures have good prognostic performance when lung adenocarcinoma patients were separated into different cohorts based on clinicopathological factors. Nine AS were selected for multivariate Cox regression analysis, namely, HNRNPLL|53258|AT, CA5B|98313|ES, MEGF6|315|ES, CDKN2A|86000|AP, BEST3|23330|AT, TTC39C|44852|AP, AP2B1|40327|AD, LETM2|83399|AT, and MKL1|62348|AP. Notably, these AS-based prognostic signatures were robustly demonstrated by survival analysis, ROC curve, and Cox regression analysis. A nomogram was generated, indicating that the consistency between predicted and actual outcome is good. Furthermore, the associated SF-AS regulatory network was identified.

The role of AS events related to TIME in lung adenocarcinoma was analyzed *via* several methods. Infiltrating stromal and immune cells consist of the primary fraction of normal cells in the tumor tissue and have a dual role during cancer onset and progression. Of note, in our study, the ESTIMATEScore in high-risk patients was lower than that in the low-risk group. Since there was not enough information on ICI treatment in the TCGA-LUAD cohort, the relationship between risk score and response to ICI treatment could not be analyzed. Risk score was positively correlated with two ICI key targets (HAVCR2 and CTLA-4) and 15 ICI-associated genes’ expression levels (i.e., CD44), which implies that risk score might contribute to tailored immunotherapy.

TIMER results showed that the AS-based risk score signature exhibited positive association with CD8+ T cells, and the proportion of CD8+ T cells is positively associated with risk score. These results indicated that high-risk samples were infiltrated by more activated cytotoxic immune cells. Therefore, the risk score correlates with anti-cancer immune response, and risk score might facilitate immunotherapy results prediction. AS regulates immune responses across a variety of conditions (17). The AS events of specific genes could influence tumor growth between tumor-immune cell interactions (18). Studies have investigated how AS serves to modulate lymphocyte activity (19). These highlight the importance of AS in the adaptive immune response to tumor.

AS events are regulated by splicing factors that are differentially expressed in cancer tissues (20). AS is increasingly described to affect the immune system, including tumor immunology (19). AS promotes tumor resistance to ICIs. Studies have demonstrated that there is a negative correlation between AS changes and somatic mutations (3, 7). For example, TDP-43 (TAR DNA binding protein, also known as TARDBP) mutation influences AS of PD-L1 pre-mRNA (21). The functional importance of PD-L1 isoforms has been demonstrated in mediating cancer immune evasion and progression (22). Changes in AS were also found in the TME (20). AS variants may be central in the interactions between tumor cells and TIME. The CD44 gene undergoes extensive AS, which generates multiple isoforms. CD44 AS-mediated positive feedback loop promotes cancer migration and invasion processes and interacts with extracellular matrix ligands (23).

The role of CDKN2A in lung adenocarcinoma has been investigated previously. CDKN2A is a tumor suppressor gene located at chromosome 9 that encodes p16 protein (24). CDKN2A inactivation is frequent in lung cancer and occurs *via* homozygous deletions, point mutations, or methylation of promoter regions (25). CDKN2A is produced by AS of E1, E2, and E3 (24). CDKN2A AS encodes for two tumor suppressors, p14ARF and p16INK4A, which positively regulate TP53 and RB1 (26). Mutant CDKN2A could regulate p16/p14 expression by AS in metastasis of renal cell carcinoma (27). Lung cancer patients with CDKN2A loss have poor overall and disease-free survival (28). In our study, we found that CDKN2A expression is negatively associated with tumor grade and ICI key genes. Collectively, lung adenocarcinoma patients with lower risk score or higher CDKN2A expression levels present more immune cells in TIME, suggesting an activated immune phenotype that results in longer overall survival. The correlation between CDKN2A expression and response to ICI treatment was also demonstrated in a recent study, highlighting the association of non-immune pathways to the outcome of ICI treatment (29). Nevertheless, further investigation is needed to explore the biological roles of CDKN2A.

A large amount of AS events is orchestrated by a limited number of SFs (30). In our study, two SFs were identified as hub SFs in the regulatory network. SFs coordinate nuclear intron/exon splicing of RNA, while SF disturbances can cause cell death. DDX39B is an RNA helicase that tethers ALY, an essential mRNA export factor, confirming the role of DDX39 in the RNA splicing/export process (31). Overexpression of DDX39B predicts poor prognosis and promotes aggressiveness of melanoma (32). DDX39B can also predict adverse efficacy of immune checkpoint therapy in clear cell carcinoma (33). DDX39B serves as a potential drug target for the treatment of androgen receptor splice variant-positive prostate cancer (34). RBM5 (RNA Binding Motif 5) has been identified as a tumor suppressor in the lung (35, 36). RBM5 regulates AS of apoptotic genes. Overexpression of RBM5 is reported to induce autophagy in human lung adenocarcinoma cells (37). The altered expression of SFs that regulate genes aberrantly spliced provides new clues to lung cancer development and drug development.

The highlight of the current study was that we proposed prognostic signatures based on AS events for monitoring the prognosis of lung adenocarcinoma patients. Our findings identified a panel of AS events that exert their biological functions in tumor immune regulation of lung adenocarcinoma. Compared with previous papers investigating AS signature in lung cancer (38, 39), this study has taken into consideration the relationship of AS prognostic signature with TIME.

However, there are several limitations. First, all data come from the TCGA database; therefore, selection bias may exist in our study. There was no independent database verification of the prognostic model due to the lack of transcriptome information in other databases. Furthermore, as our study is solely based on bioinformatics analysis, experimental validation is further warranted. Second, due to the limited data on ICI treatment, we could not confirm the ability of our signature to predict the efficacy of ICI.

In summary, integrative analyses of splicing patterns in lung adenocarcinoma were performed in our study, which was designed to strengthen prognostic scoring in lung adenocarcinoma. An AS-based prognostic nomogram was established, which could be used to predict patient survival. The comprehensive bioinformatic analyses of AS events linked the AS atlas with TIME characteristics and immune checkpoints in lung adenocarcinoma. Our study contributes to the investigation of the potential roles of AS events in the context of TIME complexity and diversity of lung adenocarcinoma. The AS-SFs regulatory network also suggests promising targets for anti-tumor therapy in lung adenocarcinoma.

## Data Availability Statement

The original contributions presented in the study are included in the article/**Supplementary Material**. Further inquiries can be directed to the corresponding authors.

## Author Contributions

ZW, LP, and ZY designed and supervised the study. ZW, LZ, YS, and LP analyzed the data and wrote the original draft. LP, JS, and GG edited the draft. All authors contributed to the article and approved the submitted version.

## Funding

This study was supported by a grant from Medical Science Research Foundation of Health Bureau of Zhejiang Province (Grant number: 2022KY545) and a grant from the Administration of Traditional Chinese Medicine of Zhejiang Province (Grant number: 2022ZA021).

## Conflict of Interest

From 2020-present JS, the Editor-in-Chief of Oncogene has sat on SABs for Vaccitech, Heat Biologics, Eli Lilly, Alveo Technologies, Pear Bio, Agenus, Equilibre Biopharmaceuticals, Graviton Bioscience Corporation, Celltrion, Volvox, Certis Oncology Solutions, Greenmantle, Zedsen, Bryologyx and Benevolent AI. He has consulted with Lansdowne partners and Vitruvian. He sits on the Board of Directors for Xerion and BB Biotech Healthcare Trust PLC. GG is Editor in Chief in Cancer Gene Therapy and the Founder and Chief Scientific Advisor of Stingray Bio.

The remaining authors declare that the research was conducted in the absence of any commercial or financial relationships that could be construed as a potential conflict of interest.

## Publisher’s Note

All claims expressed in this article are solely those of the authors and do not necessarily represent those of their affiliated organizations, or those of the publisher, the editors and the reviewers. Any product that may be evaluated in this article, or claim that may be made by its manufacturer, is not guaranteed or endorsed by the publisher.

## References

[1] Siegel RL Miller KD Fuchs HE Jemal A. Cancer Statistics, 2021. CA Cancer J Clin (2021) 71(1):7–33. doi: 10.3322/caac.21654 33433946

[2] Montes M Sanford BL Comiskey DF Chandler DS. RNA Splicing and Disease: Animal Models to Therapies. Trends Genet (2019) 35(1):68–87. doi: 10.1016/j.tig.2018.10.002 30466729 PMC6339821

[3] Climente-Gonzalez H Porta-Pardo E Godzik A Eyras E. The Functional Impact of Alternative Splicing in Cancer. Cell Rep (2017) 20(9):2215–26. doi: 10.1016/j.celrep.2017.08.012 28854369

[4] Chevrier S Levine JH Zanotelli VRT Silina K Schulz D Bacac M. An Immune Atlas of Clear Cell Renal Cell Carcinoma. Cell (2017) 169(4):736–49.e18. doi: 10.1016/j.cell.2017.04.016 28475899 PMC5422211

[5] Frankiw L Baltimore D Li G. Alternative mRNA Splicing in Cancer Immunotherapy. Nat Rev Immunol (2019) 19(11):675–87. doi: 10.1038/s41577-019-0195-7 31363190

[6] Wu HY Peng ZG He RQ Luo B Ma J Hu XH. Prognostic Index of Aberrant mRNA Splicing Profiling Acts as a Predictive Indicator for Hepatocellular Carcinoma Based on TCGA SpliceSeq Data. Int J Oncol (2019) 55(2):425–38. doi: 10.3892/ijo.2019.4834 PMC6615926 31268164

[7] Seiler M Peng S Agrawal AA Palacino J Teng T Zhu P. Somatic Mutational Landscape of Splicing Factor Genes and Their Functional Consequences Across 33 Cancer Types. Cell Rep (2018) 23(1):282–96.e4. doi: 10.1016/j.celrep.2018.01.088 29617667 PMC5933844

[8] Kouyama Y Masuda T Fujii A Ogawa Y Sato K Tobo T. Oncogenic Splicing Abnormalities Induced by DEAD-Box Helicase 56 Amplification in Colorectal Cancer. Cancer Sci (2019) 110(10):3132–44. doi: 10.1111/cas.14163 PMC6778637 31390121

[9] Lee SC Abdel-Wahab O. Therapeutic Targeting of Splicing in Cancer. Nat Med (2016) 22(9):976–86. doi: 10.1038/nm.4165 PMC5644489 27603132

[10] Zhou M Diao Z Cheng L Sun J. Construction and Analysis of Dysregulated lncRNA-Associated ceRNA Network Identified Novel lncRNA Biomarkers for Early Diagnosis of Human Pancreatic Cancer. Oncotarget (2016) 7(35):56383–94. doi: 10.18632/oncotarget.10891 PMC5302921 27487139

[11] Wang C Zheng M Wang S Nie X Guo Q Gao L. Whole Genome Analysis and Prognostic Model Construction Based on Alternative Splicing Events in Endometrial Cancer. BioMed Res Int (2019) 2019:2686875. doi: 10.1155/2019/2686875 31355251 PMC6634061

[12] Meng T Huang R Zeng Z Huang Z Yin H Jiao C. Identification of Prognostic and Metastatic Alternative Splicing Signatures in Kidney Renal Clear Cell Carcinoma. Front Bioeng Biotechnol (2019) 7:270. doi: 10.3389/fbioe.2019.00270 31681747 PMC6803439

[13] Xiao L Zou G Cheng R Wang P Ma K Cao H. Alternative Splicing Associated With Cancer Stemness in Kidney Renal Clear Cell Carcinoma. BMC Cancer (2021) 21(1):703. doi: 10.1186/s12885-021-08470-8 34130646 PMC8204412

[14] Zuo Y Zhang L Tang W Tang W. Identification of Prognosis-Related Alternative Splicing Events in Kidney Renal Clear Cell Carcinoma. J Cell Mol Med (2019) 23(11):7762–72. doi: 10.1111/jcmm.14651 PMC6815842 31489763

[15] Yoshihara K Shahmoradgoli M Martinez E Vegesna R Kim H Torres-Garcia W. Inferring Tumour Purity and Stromal and Immune Cell Admixture From Expression Data. Nat Commun (2013) 4:2612. doi: 10.1038/ncomms3612 24113773 PMC3826632

[16] Nishino M Ramaiya NH Hatabu H Hodi FS. Monitoring Immune-Checkpoint Blockade: Response Evaluation and Biomarker Development. Nat Rev Clin Oncol (2017) 14(11):655–68. doi: 10.1038/nrclinonc.2017.88 PMC5650537 28653677

[17] Chauhan K Kalam H Dutt R Kumar D. RNA Splicing: A New Paradigm in Host-Pathogen Interactions. J Mol Biol (2019) 431(8):1565–75. doi: 10.1016/j.jmb.2019.03.001 PMC7115970 30857970

[18] Wang Y Zhang H Jiao B Nie J Li X Wang W. The Roles of Alternative Splicing in Tumor-Immune Cell Interactions. Curr Cancer Drug Targets (2020) 20(10):729–40. doi: 10.2174/1568009620666200619123725 PMC8388066 32560607

[19] Martinez NM Lynch KW. Control of Alternative Splicing in Immune Responses: Many Regulators, Many Predictions, Much Still to Learn. Immunol Rev (2013) 253(1):216–36. doi: 10.1111/imr.12047 PMC3621013 23550649

[20] Brosseau JP Lucier JF Nwilati H Thibault P Garneau D Gendron D. Tumor Microenvironment-Associated Modifications of Alternative Splicing. RNA (2014) 20(2):189–201. doi: 10.1261/rna.042168.113 24335142 PMC3895271

[21] Gong B Kiyotani K Sakata S Nagano S Kumehara S Baba S. Secreted PD-L1 Variants Mediate Resistance to PD-L1 Blockade Therapy in Non-Small Cell Lung Cancer. J Exp Med (2019) 216(4):982–1000. doi: 10.1084/jem.20180870 30872362 PMC6446862

[22] Wang C Weng M Xia S Zhang M Chen C Tang J. Distinct Roles of Programmed Death Ligand 1 Alternative Splicing Isoforms in Colorectal Cancer. Cancer Sci (2021) 112(1):178–93. doi: 10.1111/cas.14690 PMC7780007 33058325

[23] Senbanjo LT Chellaiah MA. CD44: A Multifunctional Cell Surface Adhesion Receptor Is a Regulator of Progression and Metastasis of Cancer Cells. Front Cell Dev Biol (2017) 5:18. doi: 10.3389/fcell.2017.00018 28326306 PMC5339222

[24] Zhao R Choi BY Lee MH Bode AM Dong Z. Implications of Genetic and Epigenetic Alterations of CDKN2A (P16(INK4a)) in Cancer. EBioMedicine (2016) 8:30–9. doi: 10.1016/j.ebiom.2016.04.017 PMC4919535 27428416

[25] Tam KW Zhang W Soh J Stastny V Chen M Sun H. CDKN2A/p16 Inactivation Mechanisms and Their Relationship to Smoke Exposure and Molecular Features in Non-Small-Cell Lung Cancer. J Thorac Oncol (2013) 8(11):1378–88. doi: 10.1097/JTO.0b013e3182a46c0c PMC3951422 24077454

[26] Petronzelli F Sollima D Coppola G Martini-Neri ME Neri G Genuardi M. CDKN2A Germline Splicing Mutation Affecting Both P16(Ink4) and P14(Arf) RNA Processing in a Melanoma/Neurofibroma Kindred. Genes Chromosomes Cancer (2001) 31(4):398–401. doi: 10.1002/gcc.1159 11433531

[27] Sun Q Chen S Hou Y Wen X Teng X Zhang H. Mutant CDKN2A Regulates P16/p14 Expression by Alternative Splicing in Renal Cell Carcinoma Metastasis. Pathol Res Pract (2021) 223:153453. doi: 10.1016/j.prp.2021.153453 34022680

[28] Liu W Zhuang C Huang T Yang S Zhang M Lin B. Loss of CDKN2A at Chromosome 9 has a Poor Clinical Prognosis and Promotes Lung Cancer Progression. Mol Genet Genomic Med (2020) 8(12):e1521. doi: 10.1002/mgg3.1521 33155773 PMC7767555

[29] Banchereau R Leng N Zill O Sokol E Liu G Pavlick D. Molecular Determinants of Response to PD-L1 Blockade Across Tumor Types. Nat Commun (2021) 12(1):3969. doi: 10.1038/s41467-021-24112-w 34172722 PMC8233428

[30] Zhang J Manley JL. Misregulation of pre-mRNA Alternative Splicing in Cancer. Cancer Discov (2013) 3(11):1228–37. doi: 10.1158/2159-8290.CD-13-0253 PMC3823817 24145039

[31] Sugiura T Sakurai K Nagano Y. Intracellular Characterization of DDX39, a Novel Growth-Associated RNA Helicase. Exp Cell Res (2007) 313(4):782–90. doi: 10.1016/j.yexcr.2006.11.014 17196963

[32] Xing C Tian H Zhang Y Guo K Tang Y Wang Q. DDX39 Overexpression Predicts a Poor Prognosis and Promotes Aggressiveness of Melanoma by Cooperating With SNAIL. Front Oncol (2020) 10:1261. doi: 10.3389/fonc.2020.01261 32903487 PMC7435017

[33] Bao Y Jiang A Dong K Gan X Gong W Wu Z. DDX39 as a Predictor of Clinical Prognosis and Immune Checkpoint Therapy Efficacy in Patients With Clear Cell Renal Cell Carcinoma. Int J Biol Sci (2021) 17(12):3158–72. doi: 10.7150/ijbs.62553 PMC8375229 34421357

[34] Nakata D Nakao S Nakayama K Araki S Nakayama Y Aparicio S. The RNA Helicase DDX39B and Its Paralog DDX39A Regulate Androgen Receptor Splice Variant AR-V7 Generation. Biochem Biophys Res Commun (2017) 483(1):271–6. doi: 10.1016/j.bbrc.2016.12.153 28025139

[35] Jamsai D Watkins DN O’Connor AE Merriner DJ Gursoy S Bird AD. *In Vivo* Evidence That RBM5 is a Tumour Suppressor in the Lung. Sci Rep (2017) 7(1):16323. doi: 10.1038/s41598-017-15874-9 29176597 PMC5701194

[36] Sutherland LC Wang K Robinson AG. RBM5 as a Putative Tumor Suppressor Gene for Lung Cancer. J Thorac Oncol (2010) 5(3):294–8. doi: 10.1097/JTO.0b013e3181c6e330 20186023

[37] Su Z Wang K Li R Yin J Hao Y Lv X. Overexpression of RBM5 Induces Autophagy in Human Lung Adenocarcinoma Cells. World J Surg Oncol (2016) 14:57. doi: 10.1186/s12957-016-0815-7 26923134 PMC4770605

[38] Li Y Sun N Lu Z Sun S Huang J Chen Z. Prognostic Alternative mRNA Splicing Signature in Non-Small Cell Lung Cancer. Cancer Lett (2017) 393:40–51. doi: 10.1016/j.canlet.2017.02.016 28223168

[39] Zhao D Zhang C Jiang M Wang Y Liang Y Wang L. Survival-Associated Alternative Splicing Signatures in Non-Small Cell Lung Cancer. Aging (Albany NY) (2020) 12(7):5878–93. doi: 10.18632/aging.102983 PMC7185095 32282333

