# Supplementary figures and images for: A Prediction Model Using Alternative Splicing Events and the Immune Microenvironment Signature in Lung Adenocarcinoma
Source: Front Oncol. 2021 Dec 22;11:778637. doi: 10.3389/fonc.2021.778637 (PMC8728792; doi:10.3389/fonc.2021.778637)

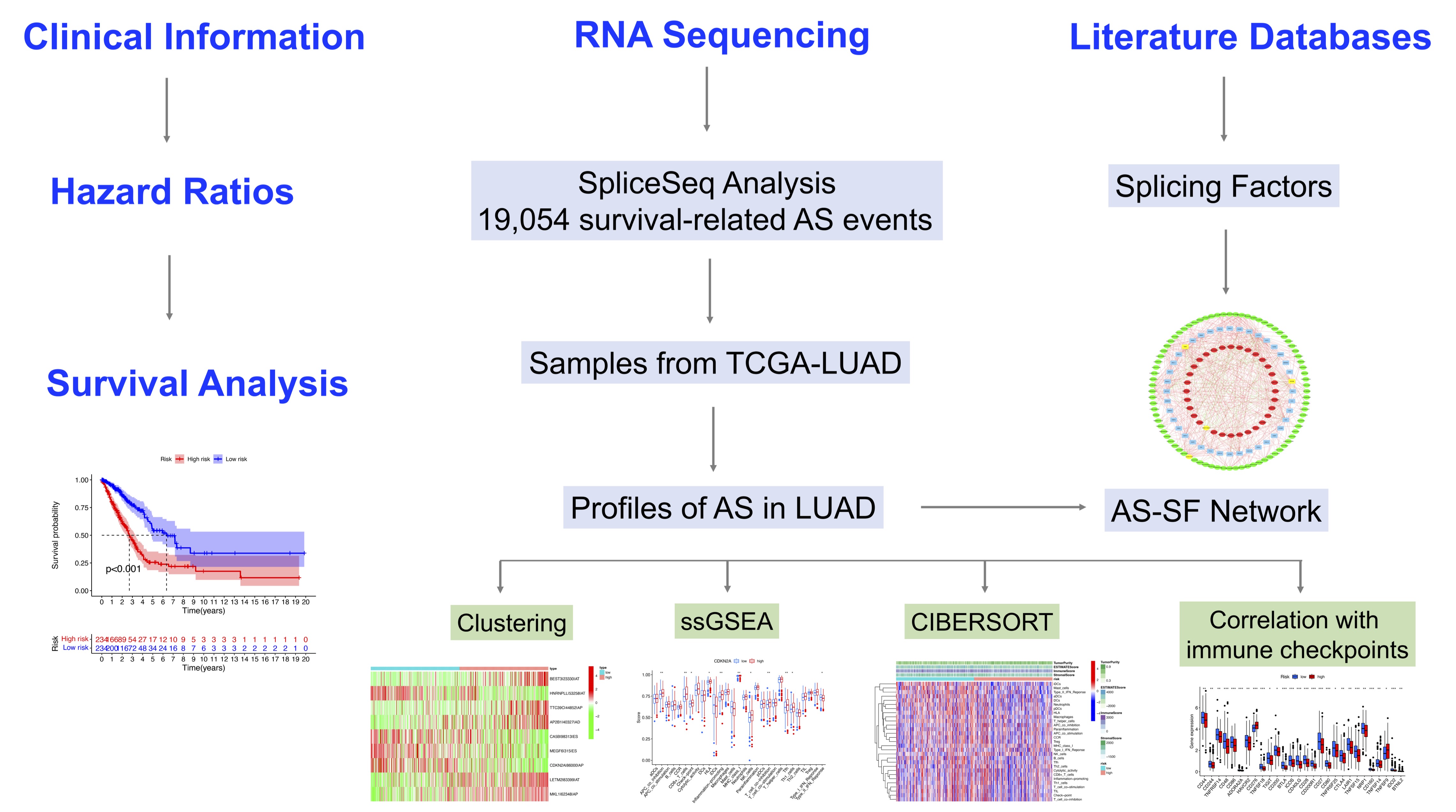

Supplement: Supplementary file 1 [file Image_1.jpeg]
